# The Impact of Complementary and Alternative Medicine on Insomnia: A Systematic Review

**DOI:** 10.7759/cureus.28425

**Published:** 2022-08-26

**Authors:** Kanika Verma, Deepeshwar Singh, Alok Srivastava

**Affiliations:** 1 Department of Yoga and Life Sciences, Swami Vivekananda Yoga Anusandhana Samsthana, Bangalore, IND; 2 Department of Panchkarma, Uttarakhand Ayurved University, Dehradun, IND

**Keywords:** yoga, valerian, kava, tai-chi, insomnia, acupressure, acupuncture

## Abstract

Insomnia is characterized by difficulty in maintaining sleep and early morning awakenings. Although pharmacotherapies and psychological interventions remain essential for conventional treatment, motivational factors and interest in using complementary and alternative therapies for insomnia have developed over the last two decades. This review aims to comprehensively explore the effects of complementary and alternative medicine (CAM) on improving sleep quality to guide evidence-based clinical decision-making and inform future research. Several electronic databases such as MEDLINE, PubMed, Scopus, EMBASE, Clinical key, Cochrane, and Research gate were explored to search the relevant articles. For the systematic review, CAM studies were classified under "manual practices," "natural practices," and "mind-body practices." A total of 35 clinical trials were selected for inclusion in the systematic review, comprising adult samples. The systematic review revealed 11 RCTs with manual practice, 12 with mind-body practice, and 12 with natural medicine practice. The methodological quality of the RCTs was measured using the modified Jadad scale, a scientific quality index of ≥ 5/10 (on the augmented Jadad scale). Effect sizes (Cohen’s d) were calculated and reported in all placebo-controlled studies with the available data. Regardless of systematic reviews, and randomized controlled trials on CAM, acupuncture, acupressure, herbal medicine, yoga, and tai chi, for insomnia, most of the RCTs did not agree with the findings. Further RCT for insomnia should be developed by considering the current advanced studies in the field of CAM.

## Introduction and background

Insomnia is identified by difficulty in maintaining sleep and early morning awakenings [1]. Consequently, it further causes workplace absenteeism, accidents, and a decline in productivity which imparts tremendous societal and economic impact [2]. One-third of the general population encounter insomnia symptoms across their lifespan [3]. Insomnia should not be confused with sleep deprivation, the former being the inability to sleep adequately, either in length or quality [4]. Most studies suggested predominance rates of insomnia disorder from 5% to 15% [5-7]. Insomnia could be a persistent issue in 31% to 75% of patients, with more than two-thirds revealing side effects for at least one year [6-8]. Due to the increasing work pressure and social challenges in an advanced society, most of the masses cannot get adequate sleep and suffer from sleep disturbance [9-12]. A detailed study shows that around 30% of adults suffer from sleep disturbance [7]. It mainly affects females and is increasing with advancing age [8]. Insomnia may be acute or chronic, and primary or secondary [1]. Primary insomnia can be defined as an individual experiencing a sleep disorder due to stress or emotions, while secondary insomnia can be due to co-morbid conditions or prior illness [1]. Insomnia has been associated with many comorbidities such as hypertension, cardiovascular disease, depression, obesity, and diabetes [2]. It can also lead to alterations in attention with episodic memory, and these cognitive impairments are clinically significant [1,2]. Hence, to maintain an individual's overall health, the treatment of insomnia is necessary [9, 10]. Conventional methods of treating insomnia generally involve either pharmacotherapies or psychological interventions [11]. The use of such kinds of drugs can cause serious adverse effects such as cognitive impairment, oversedation, daytime drowsiness, rebound discontinuation, and psychomotor disturbance [12]. In recent years, benzodiazepines (diazepam and related drugs) or nonbenzodiazepine hypnotics (zolpidem or zopiclone} have been chosen over older barbiturates which can cause death in cases of overdose. In older patients, sedating antipsychotics, e.g., olanzapine or quetiapine, and sedating anti-depressants with older tricyclic drugs, are generally regarded as "off label" [11, 12]. The new treatment guidelines evolved for benzodiazepine include low doses of sedating antipsychotics, antidepressants, and mood stabilizers [11]. Although pharmacotherapies and psychological interventions remain essential for conventional treatment, due to various motivational factors, interest in using alternative therapies and products for insomnia has developed over the last two decades.

One common treatment group used by patients with insomnia is complementary and alternative medicine (CAM) [5-7]. Research on adult insomnia patients has found that 4.5% of them practised CAM to treat their condition [9]. CAM use can be seen extensively among patients with mental disorders, commonly for managing depression or insomnia. CAM generally includes extensive therapies based on different geographical regions from various schools of thought [8]. Common CAM therapies for insomnia include herbal and nutritional medicine, acupuncture, acupressure, yoga, tai chi, and mind-body practices [10]. Mind-body interventions such as yoga help manage stress and anxiety, improving sleep quality [10]. Protein source herbal supplement L-tryptophan is also used for the treatment of insomnia [11]. Acupuncture and acupressure have been found to restore the normal sleep-wake process. They can also be employed to increase the γ-amino butyric acid content, enhancing sleep quality [12, 13]. Considering the growing public interest in CAM, these therapies and products have been researched over the past two decades to treat sleep disorders. Although few systematic reviews have been conducted on the use of acupuncture and valerian in treating insomnia, but comprehensive study on all primary CAM treatments has not been conducted. The present systematic review comprehensively explored the effects of CAM on improving sleep quality to guide evidence-based clinical decision-making and inform future research. We have systematically searched and evaluated the evidence for the impact of CAM on insomnia.

## Review

Methods

Data Sources

Several electronic databases such as MEDLINE, PubMed, Scopus, EMBASE, Clinical key, Cochrane, and Research gate were explored to search the relevant articles. The references to the articles were also examined. The search strategy was only restricted to research studies in English.

The PubMed search strategy was : (((((((((((INSOMNIA) OR (insomnia[MeSH Terms])) AND (complementary medicine[MeSH Terms])) OR (alternative medicine[MeSH Terms]))) OR (complementary and alternative medicine[MeSH Terms]))) OR Natural practices OR Manual practices OR Mind-body intervention practices OR Acupuncture OR Acupressure OR Yoga OR Tai Chi AND (The Pittsburgh Sleep Quality Index[MeSH Terms])) OR (PSQI[MeSH Terms])) OR (sleep quality[MeSH Terms])) OR (sleep latency[MeSH Terms])) OR (adjustment sleep disorder[MeSH Terms]) (((("insomnia s"[All Fields] OR "sleep initiation and maintenance disorders"[MeSH Terms] OR ("sleep"[All Fields] AND "initiation"[All Fields] AND "maintenance"[All Fields] AND "disorders"[All Fields]) OR "sleep initiation and maintenance disorders"[All Fields] OR "insomnia"[All Fields] OR "insomnias"[All Fields] OR "sleep initiation and maintenance disorders"[MeSH Terms]) AND "complementary therapies"[MeSH Terms]) OR "complementary therapies"[MeSH Terms] OR (("complementaries"[All Fields] OR "complementary"[All Fields]) AND "complementary therapies"[MeSH Terms])) AND (("Pittsburgh"[All Fields] AND ("sleep quality"[MeSH Terms] OR ("sleep"[All Fields] AND "quality"[All Fields]) OR "sleep quality"[All Fields])) AND OR "sleep quality"[MeSH Terms] OR "sleep latency"[MeSH Terms] OR "dyssomnias"[MeSH Terms]

Eligibility Criteria

Eligibility criteria have been described with the PICO framework. Inclusion and exclusion criteria for participants, intervention, comparison, and outcomes have been mentioned separately in other sections of the article.

Study Design

Randomized controlled trials reporting outcomes of the effects of CAM on insomnia and sleep quality were identified and included. Observational studies, case reports, case series, case presentations, and case-control studies were not included.

Study Participants

Regardless of health issues, adults (18 years or older) were included in the study, except for those working shifts and time zone travellers.

Interventions

For the systematic review, we have included CAM studies, classified under "manual practices," "natural practices," and "mind-body practices." All psychological and psycho-educational interventions were excluded from the review, e.g., cognitive behavioural therapy, relaxation therapy, or mindfulness (regarded as mainstream therapies). Bright-light treatment, exercise, music therapy, sensory art therapies, and aromatherapy were excluded from the study (as these therapies were not considered classical CAM interventions). Melatonin, too, was excluded from the review as the substance is a hormone, not an exogenous natural medicine. A total of eight CAM intervention RCTs met the inclusion criteria.

Comparison

For comparison, we have compared the intervention with a non-active placebo or control.

Outcome Measures

Subjective and objective sleep outcomes were evaluated, including, but not limited to, sleep quality, duration, and latency. The Pittsburgh Sleep Quality Index (PSQI) and Insomnia severity index (ISI) were used as the outcome measure in most of the studies. Duration of intervention should be ≥ 1 week.

Study Eligibility

The authors KV and AS independently screened all titles and abstracts per the inclusion and exclusion criteria. Only full-text articles that were published in English are included. Any discrepancy was resolved with discussion among other authors. The searched files were imported to the Zotero library after removing duplicate items and were freely available, and Rayyan (https:// rayyan.qcri.org), a free web-based software, was used to review articles. From the selected eligible articles, required data, including administration of intervention and control, author, year of publication, study design, follow-up, sample size, outcome measures, results, effect size and quality rating, and primary outcomes, were extracted from eligible studies.

Data Extraction

KV and AS prepared a narrative synthesis for relevant research articles, including their outcomes, variations on intervention, types, and outcomes measurement. The methodological quality assessment of the RCTs was performed using the modified Jadad scale, a scientific quality index of ≥ 5/10 (on the augmented Jadad scale) [14].

Results

**Figure 1:**
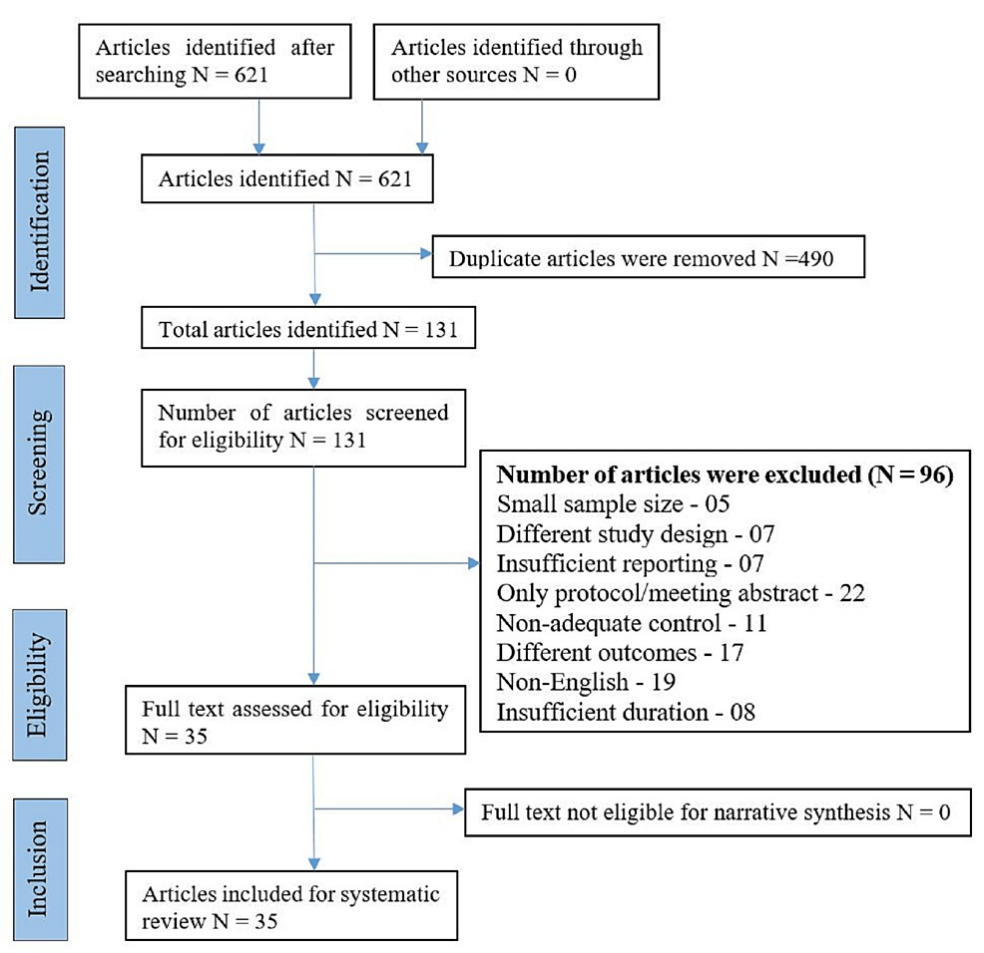
Flow Diagram (PRISMA)

KV and DS systematically searched the articles through different search engines, but as per inclusion and exclusion criteria mentioned previously, we have reviewed the articles through Rayyan (https:// rayyan.qcri.org), free web-based software to be more precise for the inclusion and exclusion criteria. As mentioned in the flow chart, among 621 identified potential studies in the field of CAM and insomnia. A total of 96 studies were removed, due to small sample size (5), different study design (7), insufficient reporting (7), only protocol/meeting abstract available (22), non-adequate control (11), different outcomes (17), non-English (19), short duration (8). This left 35 clinical trials for inclusion, primarily comprising adult samples (except for the tai chi and yoga studies, which used an older population). These CAM studies were grouped under "manual practices," "mind-body," and "natural practices." Eight CAM interventions had RCTs that met the inclusion criteria. DS and KV calculated and reported effect sizes (Cohen’s d) in all placebo-controlled studies with the available data (Figure 1).

Manual Practices

The systematic review revealed 11 RCTs with manual practice intervention [15-25]. These studies involved acupuncture (inserting fine needles to stimulate "acupoints") and acupressure (using digital or blunt pressure on "acupoints"). The trials were between two and 12 weeks, with an average sample size of 70 participants. Seven of the 11 studies measured the outcomes in the Pittsburgh sleep quality index (PSQI). The insomnia severity index (ISI) was adequate for the outcome measure in three of the 11 studies. The average quality score was 6.8 out of 10 [15-18]. Out of the four acupressure studies, they all revealed positive results on the PSQI scale with large effect sizes ranging from 1.42 to 2.12 when measured on various PSQI subscales [15-18].

Among these four acupuncture studies, one was negative, equivalent to either placebo acupuncture or basic sleep hygiene. At the same time, one was positive, equal to the positive control (clonazepam), or more effective than placebo acupuncture or positive control (estazolam). Yin et al. stated a large effect size d = 1.14 on sleep quality outcomes [19-25] (Table 1).

**Table 1:** Manual practices in the treatment of insomnia. >Statistically significant effect over control (p < 0.05). N/A, effect size could not be calculated with the data available. N/S, statistically not significant. ISI: Insomnia severity index; LSEQ: Leeds Sleep Evaluation Questionnaire; PSQI: Pittsburgh Sleep Quality Index ^a^ First author; ^b^ Intervention dosage is the total dose per day; ^c ^Effect size based on modified Cohen’s d; ^d^ A quality rating based on the modified Jadad scale; ^e^ sleep latency; ^f ^sleep duration; ^g^ sleep quality; ^h^ severity

Intervention	author^ a^	Study design ^b^	Outcome measures (Insomnia)	Result	Effect size ^c^	Qualitygrading^ d ^
Acupressure	Yeung (2018)[15]	Two-arm RCT, n = 31, eight weeks	ISI	Self-administered acupressure group had a significantly lower ISI score than the subjects in the sleep hygiene education group	0.56	7/10
Acupressure	Zheng (2014)[16]	Two-arm RCT, n = 75, four weeks	PSQI	Acupressure improves sleep quality more than control	0.64	7/10
Acupressure	Nordio (2008) [17]	Three-arm RCT, n = 44, three weeks	PSQI	Acupressure is more effective than sham acupressure after three weeks on PSQI total score	1.91^h^	9/10
Acupressure	Chen(1999) [18]	Three-arm RCT, n = 84, four weeks acupressure vs. sham acupressure vs. Control (not identified)	PSQI	Acupressure is more effective than sham acupressure and controls on PSQI total score, latency, and duration sub-scores	2.12^e^ 1.66^f^ 1.52^g^ 1.42^h^	8/10
Acupuncture	Bosch (2013) [19]	Five-arm RCT, n=40, three months	PSQI	Acupuncture is more effective than control to improve sleep in both schizophrenia and depression patients	1.09	7/10
Acupuncture	Wang (2020)[20]	Three-arm RCT, n=129, five weeks single acupuncture vs. multi acupuncture vs. sham control	PSQI and Athens Insomnia Scale questionnaire	PSQI scores were significantly decreased in single and multi- acupuncture groups compared to the controlled group.	0.88	6/10
Acupuncture	Wang (2021) [21]	Two-arm RCT, n=82, three weeks	PSQI and ISI	PSQI scores and ISI scores were decreased in the acupuncture group than in the sham-controlled group.	0.02	8/10
Acupuncture	Yin (2017) [22]	Two-arm RCT, n=72, four weeks,	ISI	Acupuncture treatment is more effective than sham acupuncture treatment in increasing sleep quality in insomnia patients	1.14	6/10
Acupuncture	Xuan (2007) [23]	Two-arm RCT, n=46, acupuncture vs. oral administration of estazolam in control group	PSQI	Estazolam was better than acupuncture treatment in prolonging sleeping time. Acupuncture treatment was better than the control group in the improvement of somnipathy and the increase of daytime functional state	N/A	8/10
Acupuncture	Wang (2008) [24]	Three-arm RCT, n = 44, two-week abdominal acupuncture, seven treatments + placebo pill vs estazolam + sham acupuncture estazolam group)	LSEQ	Acupuncture is more effective than estazolam and sham acupuncture on LSEQ total score	N/A	7/10
Acupuncture	Da Silva (2005) [25]	Two-arm RCT, n = 30, eight weeks (pregnant women) acupuncture 8-12 treatments vs. sleep hygiene advice	Purpose-designed self-report sleep questionnaire	Acupuncture and sleep hygiene advice affects sleep quality equally after eight weeks. Only effective at follow-up two weeks after stopping acupuncture (10 weeks)	N/A	7/10

Mind-Body Practices

There were 12 mind-body intervention RCTs of sufficient methodological rigour in this review [26-37]. The trial length was between four and 24 weeks, with an average sample size of 90 participants. Five out of the seven studies used PSQI as the outcome measure. The average rating of the quality of the studies was eight out of 10. The yoga intervention studies revealed a positive effect on wait-list control. Manjunath et al. and Mustian et al. studies found large effect sizes (d = 1.52) and d = 2.56 on sleep quality outcome measures [31, 32]. One study (Ward et al.) found no significant difference between yoga and control groups [30].

All five tai chi trials were positive on various PSQI outcome measures compared with health education or low-impact exercise [33-37]. Large effect sizes prevailed in sleep duration d = 2.15 and sleep quality d = 1.05 in Li et al. [35]. At the same time, a marked divergence was observed in Irwin et al. with a large effect size on sleep severity (global score) d = 1.57 compared to small effect sizes on sleep duration d = 0.22, and sleep quality d = 0.44 [37] (Table 2).

**Table 2:** Mind-body intervention practices in the treatment of insomnia. >Statistically significant effect over control (p < 0.05). N/A, effect size could not be calculated with the data available. N/S, statistically not significant. CASIS: Asthma and COPD Sleep Impact Scale; PSQI: Pittsburgh Sleep Quality Index; ISI: Insomnia severity index ^a ^First author; ^b ^Intervention dosage is the total dose per day; ^c ^Effect size based on modified Cohen’s d; ^d^ A quality rating based on the modified Jadad scale; ^e^ sleep latency; ^f^ sleep duration, ^g^ sleep quality, ^h^ severity

Interventions	Author ^a^	Study design ^b^	Outcome measures (insomnia)	Result	Effect size ^c ^	Qualitygrading ^d^
Yoga	Özer (2021) [26]	Two-arm RCT, n = 60, eight weeks Yoga vs. Control	CASIS	Yoga improves sleep in comparison to control	N/A	7/10
Yoga	Susanti (2022) [27]	Two-arm RCT, n = 104, 20 weeks; yoga vs. control	PSQI	Yoga improved sleep quality significantly in postmenopausal and perimenopausal women in comparison to the control	N/A	6/10
Yoga	Ghaffarilaleh (2019) [28]	Two-arm RCT, n = 62, 10 weeks; yoga vs. control	PSQI	Yoga helps in improving the quality of sleep, sleep latency and sleep efficiency in patients with premenstrual syndrome in comparison to the control	0.26	6/10
Yoga	Guerra (2020) [29]	Two-arm RCT, N= 64, eight weeks; yoga vs. Control	PSQI	Significant correlation between sleep and yoga; sleep latency was lower in the yoga group	NA	7/10
Yoga	Ward (2017) [30]	Two-arm RCT, n=26, nine weeks; yoga vs. control	Visual analogue scale, sleep quality, measured using the seven‐item ISI, Health Assessment Questionnaire Disability Index	No significant difference between both groups	0.06	7/10
Yoga	Manjunath (2005) [31]	Three-arm RCT, n =70, six-month (older population) yoga vs. herbal medicine vs. waitlist control	Sleep questionnaire (purpose-designed): Determining latency, waitlist, and herbal medicine formula on sleep quality, total sleep, subsequent day effects, and total latency	Yoga during the six-month evaluation was superior to the waitlist and herbal medicine formulas on sleep latency, full sleep, and “feeling of being rested.”	1.20^e^, 1.02	8/10
Yoga	Mustian (2013) [32]	Two-arm RCT, n=410, four weeks; yoga vs. control	PSQI	Yoga intervention consists of pranayama (breathing exercises), gentle Hatha and restorative yoga asanas, and meditation > control	2.56	8/10
Tai chi	Siu (2021) [33]	Three-arm RCT, n = 320, 12 weeks; tai chi training vs. exercise vs. control groups	PSQI and ISI	Tai chi training groups significantly reduced the PSQI scores in older adults compared to exercise and control group	0.65, 0.55	6/10
Tai chi	Nguyen (2012) [34]	Three-arm RCT, n = 102, six months; tai chi vs. control	PSQI	Tai chi is effective in improving sleep quality in community-dwelling elderly compared to control	0.26	6/10
Tai chi	Li (2004) [35]	Two-arm RCT, 24-week, n = 118 (older adults); tai chi vs. low-impact exercise	PSQI	Tai chi was more effective than low-impact exercise in increasing sleep duration and improving reported sleep quality, sleep latency, and sleep efficiency	2.15^b^, 1.05^c^	8/10
Tai chi	McQuade (2017) [36]	Three-arm RCT, n=90, one and three months tai chi vs. light exercise vs. waitlist control	PSQI	Tai chi, light exercise and waitlist control were equally effective in reducing sleep disturbance for those undergoing radiotherapy for prostate cancer	1.63, 1.23	6/10
Tai chi	Irwin(2008) [37]	25-weeks RCT, n = 112 (older adults) tai chi vs. control	PSQI: sleep quality (primary outcome)	Tai chi had a significant effect on sleep quality, severity, and duration outcomes compared to control	0.22^f^ , 0.44^g^, 1.57^h^	8/10

Natural medicine practices

The review identified 12 RCTs of sufficient methodological rigour [38-48]. Three studies included kava and valerian interventions. Six studies included valerian intervention [38-40]. Three studies included tryptophan intervention [13,47,48]. The trials were from two to eight weeks (commonly two to four weeks) with an average sample size of 150 participants. The herbal medicine kava met the inclusion criteria compared to the placebo. The analysis of Lehrl et al.'s study showed a benefit for kava over placebo on the quality of sleep outcome [40]. The valerian studies exhibited diversified results, with three positive (more effective than placebo and equivalent to oxazepam) and three negative results (equal to placebo) [41-46]. For most of the studies, effect size calculations could not be available. For L-tryptophan trials, the average quality rating was seven out of 10, while herbal medicines studies showed a higher rating of 8 out of 10. Among the three L-tryptophan studies, two were positive on several outcomes. Hudson et al., in their analysis, mentioned a large effect size on increased sleep duration d = 1.16, and a small effect size on sleep quality d = 0.28 [13] (Table 3).

**Table 3:** Natural medicine practices in the treatment of insomnia >Statistically significant effect over control (p < 0.05). N/A, effect size could not be calculated with the data available. N/S, statistically not significant. PSQI: Pittsburgh Sleep Quality Index; ISI: insomnia severity index; VAS: visual analogue scales ^a^ First author; ^b ^Intervention dosage is the total dose per day; ^c^ Effect size based on modified Cohen’s d; ^d^ A quality rating based on the modified Jadad scale; ^e^ sleep latency; ^f ^sleep duration; ^g^ sleep quality; ^h ^severity

Interventions	Author ^a^	Study design ^b^	Outcome measures (insomnia)	Result	Effect size ^c^	Qualitygrading ^d^
Kava and Valerian	Wheatley (2001) [38]	RCT, n=24, crossover trial, six weeks	VAS, Wheatley Stress Profile; time to fall asleep, hours slept, and mood on final waking.	Kava, valerian and placebo groups had no significant difference	N/A	7/10
Kava and Valerian	Jacobs(2005) [39]	Three-arm RCT, n = 391, four weeks	Insomnia Severity Index (ISI): overall score, sleep latency, outcome measures number of awakenings	Neither kava nor valerian relieved anxiety or insomnia more than the placebo	0.02^e^	9/10
Kava and Valerian	Lehrl (2004) [40]	Three-arm RCT, n = 61, four weeks	Görtelmeyer Sleep Questionnaire, quality of sleep SCALE	Kava is more effective than placebo in improving the quality of sleep at the week four endpoint	N/A	8/10
Valerian	Zare (2021) [41]	Two-arm RCT, n=72, four weeks	PSQI, the prothrombin time (PT), and partial thromboplastin time (PTT)	No significant difference between both groups	N/A	8/10
Valerian	Oxman (2007) [42]	Two-arm RCT, n = 405, two weeks	Internet sleep diary: sleep onset latency, quality awakenings, assessment; sleep diary quality	Valerian is more effective than placebo on sleep quality	N/A	9/10
Valerian	Coxeter (2003) [43]	Two-arm RCT, n = 42, six weeks	Sleep diary: sleep latency, self-rated outcomes were ‘poor’ or modest	Valerian had no significant difference from placebo	N/A	9/10
Valerian	Ziegler (2002) [44]	Two-arm RCT, n = 202, six weeks	Görtelmeyer Sleep outcome; sleep quality, and on all other subscale outcomes	Valerian is more effective than control	N/A	8/10
Valerian	Koetter (2007) [45]	Two-arm RCT, n = 30, four weeks; valerian-valerian vs. placebo	Sleep monitoring device: sleep awakenings, efficiency; REM sleep stages; Clinical Global Assessment	Sleep latency with valerian-hops and placebo groups had no difference	N/A	6/10
Valerian	Morin (2005) [46]	Three-arm RCT, n = 184, four weeks	Sleep diary: subjective sleep efficacy, total sleep time, Insomnia Severity Index	Valerian-hops reduced insomnia	0.81^h^	8/10
Tryptophan	Hudson (2005) [13]	Three-arm RCT, n = 57, three weeks 250 mg tryptophan food vs. 250 mg pharmaceutical tryptophan vs. placebo	Sleep diary: sleep efficiency, quality, total, awakening time	Tryptophan food and pharmaceutical tryptophan were more effective than placebo on all outcomes	1.16^ f^, 0.28^g^	8/10
Tryptophan	Demisch (1987) [47]	RCT crossover, n = 39, eight weeks 2 g tryptophan vs. 0.04 g tryptophan scale) placebo	Sleep quality scale (1-5 Likert scale)	2 g tryptophan was more effective than 0.04 g tryptophan across groups in phase 1 and within subjects in Group A	N/A	6/10
Tryptophan	Hartmann (1983) [48]	Four-arm RCT, n = 96, two weeks	Various outcome scales	Sleep latency had a significant difference after treatment with tryptophan	N/A	6/10

A review of all quality studies suggested that CAM may have the potential to improve sleep quality in a variety of patient populations. Although evidence is limited, this systematic review, which includes studies published till Jan. 2022, provides evidence that CAM may be useful for the treatment of both uncomplicated insomnias as well as insomnia co-morbid conditions.

Despite the substantial clinical trial literature, several studies were excluded due to methodological shortcomings. Only 35 English language RCTs met the inclusion criteria. In various RCTs of herbal medicine, mostly involving valerian, researchers employed a short study duration and small sample size, restricting the study's statistical power. Moreover, several acupuncture studies had an 'active' control group, mostly involving another type of acupuncture.

Many studies could not calculate the effect size due to negative results or insufficient data (e.g., no standard deviation). As a result, the effect sizes noted in the positive studies (in natural medicine and manual practices) should be tempered concerning the negative studies. Quality grading of RCTs has been displayed in respective tables of manual, mind body and natural medicine practice. The majority of RCTs for manual practice scored 7/10 for quality of grading. Most RCTs for mind-body practices scored 6/10 while assessing quality grading. Mostly, RCTs with natural medicine practice scored 8/10 score for quality grading.

Discussion

Findings revealed that the evidence for natural medicine practices in treating insomnia was also conflicting. Valerian was one of the most studied soporific natural medicines for its rich folkloric tradition of use in conditions of restlessness, hysteria, headache, nervousness, and mental depression. As detailed in Table 3, the evidence regarding valerian was quite mixed and did not support its use in treating insomnia. These results follow the systematic reviews and meta-analyses done by Bent et al. and Taibi et al. [49, 50]. The study of Bent et al., which included 16 eligible RCTs on valerian and valerian in combination with other herbal medicines, suggests that nine out of 16 studies did not have positive outcomes concerning the improvement of sleep quality [49]. The Taibi et al. review, which included 29 controlled studies, consistently stated that most studies lacked any significant difference between valerian and placebo. Valerian, combined with hops or kava, did not seem to support the available data. Kava may provide a prospective alternative for managing insomnia [50]. However, Lehrl's studies had a different opinion, and presently as kava is withdrawn in several jurisdictions, further studies about its safety and efficacy are much needed [40].

L-tryptophan, an exogenous amino acid converted into serotonin, has been widely studied in treating insomnia and depression [51]. However, the results were encouraging but varied concerning different sleep outcomes. The studies on various animals and humans consistently suggest that L-tryptophan increases sleepiness and decreases sleep latency [51, 52]. It has been observed that the best results seem to occur in cases of mild insomnia with long sleep latency and the absence of any medical or psychiatric comorbidity [51, 52]. A study by Hudson et al. determined a large effect size on increased sleep duration and a negligible effect on sleep quality [13]. A survey by Irwin et al. consistently revealed a substantial effect size for tai chi in reducing insomnia severity. In contrast, sleep duration and quality effects had poor clinical outcomes [37]. The heterogeneous nature of samples throughout the studies made these effect size differences [37]. 

Acupuncture and acupressure seem to contribute to treating insomnia, probably by the neurochemical modulatory activity of serotonin, dopamine, and endogenous opioids [53]. The review by Cheuk et al. concluded with seven rigorous methodological trials that acupuncture and acupressure help improve sleep quality scores. Still, the evidence for acupuncture as a hypnotic intervention was inconsistent. Compared to control or no treatment, the efficacy of acupuncture and its variants was inconsistent among studies, including many sleep parameters, such as sleep onset latency, time to wake after sleep onset, and total sleep duration [53]. Further, Yeung et al. revealed that definitive conclusions could not be derived on acupuncture's efficacy for insomnia. This conclusion was reached due to the methodological quality of RCTs as the limitations of the study designs hampered studies. For instance, publications of such studies have also provided limited information about inclusion/exclusion criteria, outcomes measured, missing baseline data, randomization methods, and the specific acupuncture approach [54].

Mind-body practices such as yoga and tai chi in insomnia and different sleep disorders are enhancing popularity, particularly in the ageing population who might prefer low-effect exercises [35]. Comparatively, findings supported the benefits of exercise in improving sleep quality and reducing the severity of insomnia in older people. However, a Li et al. study compared tai chi to low-effect exercise and found it superior to low-effect exercises in all outcomes [35]. While Manjunath et al., the yoga study included older participants with sleep issues and noted yoga was superior to both wait-list and herbal medicine on sleep latency and total sleep [31]. Mind-body practices comprise multicomponent interventions, considered to give rise to similar physiological processes to traditional relaxation methods, which have been investigated as treatment options for insomnia [26-37]. Mechanistically, yoga acutely affects the activity of the autonomic nervous system and may decrease the gamma-aminobutyric acid levels and inflammatory markers. Probably by neurobiological pathways, yoga may improve sleep quality [28, 29]. Data from small RCTs suggested that yoga improves subjective and objective sleep quality, reducing insomnia symptoms in adults with chronic medical conditions [26, 28, 29, 31]. One of the largest RCTs of Yoga illustrated reduced hypnotic medication use in cancer survivors with sleep disturbance by 21% in the yoga group compared with 5% in the control group [32]. However, several emerging clinical trials on yoga found that most participants faced a general sleep disturbance. Moreover, yoga studies including pranayama, breathing exercises, gentle hatha, restorative yoga asanas, and meditation assessing sleep outcomes showed common methodological limitations of sample sizes and limited use of objective outcome measures [27-32]. Further studies on yoga are encouraged with participants with a confirmed diagnosis of insomnia using validated sleep assessment scales.

Similarly, in studies for tai chi intervention, the focus was more on sleep quality rather than insomnia. Several RCTs suggested improvement in reported sleep quality, with tai chi intervention, particularly among older adults. Therefore, tai chi may improve sleep quality in different populations, specifically older adults [33-37]. Its impact on objective measures in chronic insomnia needs further explained.

In reference to data from the United States National Health Interview Survey, 17.4% of adults (n= 93 386) reported insomnia or regular sleep disturbance, and 4.5% used CAM therapies to improve their sleep quality. 56% of the individuals said that CAM was essential to maintaining their overall health and well-being, while 72% observed that CAM improved insomnia disorders significantly. Younger and highly qualified persons believe in CAM to improve insomnia symptoms [55]. It has been noticed that traditional Indo-Asian therapies such as acupuncture, acupressure, and yoga were more widely studied compared to standard Western CAM therapies. The broadly researched herbal medicine is valerian in the form of monotherapy or combination with hops or kava [13, 15-48]. Future research in other herbal medications or Western CAM therapies with potential hypnotic effects is recommended as current research in these areas is insufficient.

Strength and limitations

The strengths of this systematic review included the search and synthesis of all relevant studies across several databases, the rigorous methodological inclusion criteria, and a quality assessment of all clinical trials with calculations of the effect size of studies.

This review also had some limitations. These were language constraints, as we excluded non-English publications; several valerian studies were published in German, and acupuncture studies were published in Chinese. Secondly, it lacks appropriate clinical trials with methodological weakness, for instance, adequate sample sizes. While reporting complete data, the long-term efficacy and safety of CAM interventions should have been employed.

## Conclusions

This systematic review revealed that CAM interventions might benefit various populations with insomnia and a range of sleep measures. However, clinically relevant conclusions cannot be drawn because of clinical heterogeneity and methodological limitations. Regarding CAM treatments for insomnia, it demonstrates evidence in support of acupressure, yoga, and tai chi; mixed evidence for the use of acupuncture and L-tryptophan; and insufficient evidence for the practice of natural medicines such as valerian. More RCTs with rigorous research design covering a more comprehensive range of CAM interventions with particular consideration for long-term safety and potential side effects are needed to establish the impact of CAM on insomnia, as well as the potential of CAM to be used in interventions for populations with various health conditions or specific demographic groups. Clinical trials exploring the use of CAM adjuvantly with conventional therapies like exercise, psychological interventions, or pharmacotherapies may also promise to improve sleep outcomes and provide clinically relevant evidence. Ultimately, the biological mechanism by which CAM improves sleep quality, and insomnia disorder should be studied to understand the techniques such as yoga and tai chi work on insomnia, in the presence of various health conditions.

## Appendices

PubMed Search strategy

The PubMed search strategy was : (((((((((((INSOMNIA) OR (insomnia[MeSH Terms])) AND (complementary medicine[MeSH Terms])) OR (alternative medicine[MeSH Terms]))) OR (complementary and alternative medicine[MeSH Terms]))) OR Natural practices OR Manual practices OR Mind-body intervention practices OR Acupuncture OR     Acupressure OR Yoga OR Tai Chi  AND (The Pittsburgh Sleep Quality Index[MeSH Terms])) OR (PSQI[MeSH Terms])) OR (sleep quality[MeSH Terms])) OR (sleep latency[MeSH Terms])) OR (adjustment sleep disorder[MeSH Terms]) (((("insomnia s"[All Fields] OR "sleep initiation and maintenance disorders"[MeSH Terms] OR ("sleep"[All Fields] AND "initiation"[All Fields] AND "maintenance"[All Fields] AND "disorders"[All Fields]) OR "sleep initiation and maintenance disorders"[All Fields] OR "insomnia"[All Fields] OR "insomnias"[All Fields] OR "sleep initiation and maintenance disorders"[MeSH Terms]) AND "complementary therapies"[MeSH Terms]) OR "complementary therapies"[MeSH Terms] OR (("complementaries"[All Fields] OR "complementary"[All Fields]) AND "complementary therapies"[MeSH Terms])) AND (("Pittsburgh"[All Fields] AND ("sleep quality"[MeSH Terms] OR ("sleep"[All Fields] AND "quality"[All Fields]) OR "sleep quality"[All Fields])) AND OR "sleep quality"[MeSH Terms] OR "sleep latency"[MeSH Terms] OR "dyssomnias"[MeSH Terms]

Cochrane search strategy

#1        insomnia: 12861

#2        MeSH descriptor: [Sleep Initiation and Maintenance Disorders] explode all trees: 2818

#3        complementary and alternative medicine: 5768

#4        complementary and alternative therapy: 4452

#5        complementary therapy: 9327

#6        alternative therapy: 32303

#7        MeSH descriptor: [Complementary Therapies] explode all trees: 22054

#8        MeSH descriptor: [Complementary Therapies] explode all trees: 22054

#9        alternative medicine: 18452

#10      complementary medicine: 9033

#11      Pittsburgh Sleep Quality Index: 3640

#12      PSQI: 2516

#13      MeSH descriptor: [Sleep Quality] explode all trees: 80

#14      sleep duration: 8051

#15      sleep latency: 3033

#16      MeSH descriptor: [Sleep Latency] explode all trees  20

#17      #1 OR #2 AND #3 OR #4 OR #5 OR #6 OR #7 OR #8 OR #9 AND #10 OR #11 OR #12 OR13 OR #14 OR #15 OR #16: 77959

#18         #1 OR #2 AND #5 OR #6 AND #11: 12969
